# Utility and limitations of EEG in the diagnosis and management of *ALDH7A1*-related pyridoxine-dependent epilepsy. A retrospective observational study

**DOI:** 10.3389/fneur.2024.1355861

**Published:** 2024-02-14

**Authors:** Vibeke Arntsen, Ahmed Jamali, Alma Sikiric, Erle Kristensen, Trine Tangeraas, Guste Kupliauskiene, Sigurbjörg Stefansdottir, Laurence A. Bindoff, Trond Sand, Eylert Brodtkorb

**Affiliations:** ^1^Department of Neurology and Clinical Neurophysiology, St. Olav University Hospital, Trondheim, Norway; ^2^Department of Neuromedicine and Movement Science, Norwegian University of Science and Technology, Trondheim, Norway; ^3^Kavli Institute for Systems Neuroscience, Center for Computational Neuroscience, Norwegian University of Science and Technology, Trondheim, Norway; ^4^Department of Neurohabilitation, Oslo University Hospital, Oslo, Norway; ^5^Department of Medical Biochemistry, Oslo University Hospital, Oslo, Norway; ^6^Department of Clinical Medicine (K1), University of Bergen, Bergen, Norway; ^7^Norwegian National Unit for Newborn Screening, Division of Paediatric and Adolescent Medicine, Oslo University Hospital, Oslo, Norway; ^8^Department of Paediatric and Adolescent Medicine, Stavanger University Hospital, Stavanger, Norway; ^9^Department of Neurology and Clinical Neurophysiology, Stavanger University Hospital, Stavanger, Norway; ^10^Department of Neurology, Haukeland University Hospital, Bergen, Norway

**Keywords:** pyridoxine, epilepsy, *ALDH7A1*, EEG, burst suppression

## Abstract

**Purpose:**

Pyridoxine-dependent epilepsy due to *ALDH7A1* variants (PDE-ALDH7A1) is a rare disorder, presenting typically with severe neonatal, epileptic encephalopathy. Early diagnosis is imperative to prevent uncontrolled seizures. We have explored the role of EEG in the diagnosis and management of PDE.

**Methods:**

A total of 13 Norwegian patients with PDE-ALDH7A1 were identified, of whom five had reached adult age. Altogether 163 EEG recordings were assessed, 101 from the 1st year of life.

**Results:**

Median age at seizure onset was 9 h (IQR 41), range 1 h-6 days. Median delay from first seizure to first pyridoxine injection was 2 days (IQR 5.5). An EEG burst suppression pattern was seen in eight patients (62%) during the first 5 days of life. Eleven patients had recordings during pyridoxine injections: in three, immediate EEG improvement correlated with seizure control, whereas in six, no change of epileptiform activity occurred. Of these six, one had prompt clinical effect, one had delayed effect (< 1 day), one had no effect, one had uncertain effect, and another had more seizures. A patient without seizures at time of pyridoxine trial remained seizure free for 6 days. Two patients with prompt clinical effect had increased paroxysmal activity, one as a conversion to burst suppression. Autonomic seizures in the form of apnoea appeared to promote respiratory distress and were documented by EEG in one patient. EEG follow-up in adult age did not show signs of progressing encephalopathy.

**Conclusion:**

A neonatal burst suppression EEG pattern should raise the suspicion of PDE-ALDH7A1. Respiratory distress is common; isolated apnoeic seizures may contribute. EEG responses during pyridoxine trials are diverse, often with poor correlation to immediate clinical effect. Reliance on single trials may lead to under-recognition of this treatable condition. Pyridoxine should be continued until results from biomarkers and genetic testing are available.

## 1 Introduction

Pyridoxine-dependent epilepsy (PDE) with *ALDH7A1* gene variants (PDE-ALDH7A1) is a rare autosomal recessive etiology-specific developmental and epileptic encephalopathy (DEE) (1). Classical PDE presents with severe neonatal seizures, but the clinical presentation is heterogeneous, and in atypical forms, onset may also occur in later childhood and even in adolescence (2). PDE was first discovered serendipitously in 1954 (3), and the disorder was defined by response to pyridoxine (PN) alone until the underlying genetic cause was identified in 2006 (4). Characteristic biochemical markers involved in lysine degradation and rapid genetic testing are now widely available for diagnostic confirmation (2, 5, 6). Timely diagnosis and treatment with PN are imperative to prevent uncontrolled seizures and status epilepticus, which may hamper neurocognitive development. Moreover, early supplementary dietary lysine restriction has shown beneficial effects (5, 7, 8).

The spectrum of EEG features in PDE-ALDH7A1 has been the focus of several studies. EEG abnormalities during the neonatal period are variable and comprise focal and multifocal epileptiform activity with unilateral and bilateral distribution (9–11). Ictal discharges may occur without clinical correlates (2, 12). Burst suppression patterns are common, but this finding is non-specific and sometimes absent (11, 13–15). The EEG response to PN has been described in detail by various authors. Attenuation of epileptiform activity as well as amplitude flattening have been described as characteristic features (9), but such changes also occur in non-PDE infantile seizures (16, 17). Most publications focussing on EEG findings only include a limited number of patients, usually with short follow-up periods, and several studies took place prior to the advent of biomarkers and genetic analyses for validation of true PDE-ALDH7A1 (9, 13, 16). The subsequent development of EEG changes in adolescents and adults has only rarely been reported (10).

We have recently described the phenotypic variety and the clinical course of 13 Norwegian patients with neonatal-onset PDE-ALDH7A1 (18). The aim of the present study was to explore specifically the role of EEG in the diagnosis and management of patients within this nationwide cohort spanning the age spectrum from neonates to adults.

## 2 Materials and methods

### 2.1 Patients

As reported in our previous study on PDE (18), patients were identified by contacting all Norwegian, neurological, pediatric and neurohabilitation departments as well as relevant professional societies. A total of 13 Norwegian patients with PDE-ALDH7A1 aged 2 months (mo) to 55 years (y) were included. None of them were born preterm. Five subjects had achieved adult age (Table 1). As previously reported, the median age at seizure onset was 9 h (h) (IQR 41 h), range 1 h-6 days after birth (18). All were genetically confirmed, apart from Patient 3, who had a similar clinical picture as his younger brother (Patient 10) but remained untreated and died from status epilepticus at age 10 mo. Seven children had received dietary lysine restriction/arginine enrichment.

**Table 1:** EEG recording types in 13 patients with PDE-ALDH7A1.

	**No of EEGs**	**No of patients**
Total	163	13
Recordings 1st year of life	101	13
Standard recordings	138	13
aEEG	3	3
Sleep deprived	4	4
LTM	18	7
Available digital recordings	78	10
Paper recordings	48	5
Reports	37	4

aEEG, amplitude integrated EEG; LTM, long-term monitoring.

Seizure types were classified by the predominant clinical features described in medical records using the modified classification of neonatal seizures by the International League Against Epilepsy (ILAE). All neonatal seizures are considered to be exclusively of focal origin (19).

### 2.2 EEG

We collected information from available EEG recordings covering the period from 1968 to 2023. Altogether, 163 recordings were assessed, including 101 taken during the 1st year of life (Table 1). The number of EEGs per patient ranged from 2 to 21 (median 14). The earliest EEG recordings were performed within 1–15 days, with nine recordings from the first 1–3 days of life and four from age 5–15 days. EEG follow up range was 2 mo-55 y (median 3 y, 3 mo).

All available EEG recordings were extracted and reviewed along with relevant clinical, neuroimaging and genetic data. We studied the initial EEG characteristics including the responses to PN treatment and the evolution of EEG findings with age.

Burst suppression (BS) was defined according to the American Clinical Neurophysiological Society guidelines and the International Federation of Clinical Neurophysiology glossary of terms as invariably abnormal EEG bursts devoid of normal graphoelements separated by prolonged and abnormally low voltage periods < 5–10 μV (20, 21). Abnormal alternating patterns with prolonged interburst intervals or voltage depressed for postmenstrual age were categorized as “excessive background discontinuity” (20), whereas transient BS patterns and tracé alternant, which occur in quiet sleep of normal newborns were excluded. Asymmetry was defined as amplitude difference exceeding 50%. Asynchrony implied a temporal delay >1.5 to 2 s between the bursts of identical waveforms over both hemispheres (20, 22).

EEG elements were classified according to the Standardized computer-based organized reporting of EEG (SCORE) and international glossaries of terms (20–23).

### 2.3 Ethics

The study was approved by the Regional Ethical Committee of Mid-Norway (No. 200284).

## 3 Results

### 3.1 Seizure semiology and clinical features

The most commonly reported seizure type in the neonatal phase was classified as sequential seizures with tonic components. Clonic and myoclonic seizures were also described in most patients and oral automatisms occurred in several (Table 2).

**Table 2:** Overview of demographics, genetic variants, clinical data, and EEG features.

**Patient number^a^**	**Sex^a^**	**Age at last follow-up^a^**	**Genetic variant ALDH7A1 (NM_001182.4)^a^**	**Seizure onset age^a^**	**First EEG pattern**	**Early ictal events**			**EEG at last follow up (age)**
						**Seizure types**	**ECS/EGS-only**	**Clinical-only**	
1	F	2 mo	c.1279G>C, p.(Glu427Gln) homozygous	1 h	ED bil (1 d)	SequentialTonicClonic	ECS (2 h)	+ (2 d)	NA
2	M	3 mo	c.1279G>C, p.(Glu427Gln) homozygous	1 h	BS (1 d), synch, symm	SequentialTonicSpasms?	ECS, EGS-only (1 d)	+ (1 d)	Normal (10 d)
3	M	10 mo at death	Likely same as the younger brother, Patient 10	3 h	BS^*b*^ (3 d)	SequentialMyoclonicTonicClonicAutomatisms	ECS (Ictal BS), EGS-only < 10 s (BRD) (3 d)	+ (3 d)	Cont ED/SE (10 mo)
4	F	18 mo	c.1008+1G>A homozygous	1 h	BS (2 d), synch, symm	SequentialTonicMyoclonicClonicBehavioral arrest?	EGS-only (3 d) ECS, EGS-only (5 d)	+ (3 d, 5 d)	Mild ISBA (1.5 y)
5	F	2 y, 3 mo	c.1279G>C, p.(Glu427Gln) homozygous	22 h	BS (1 d), synch, symm	SequentialMyoclonicTonicAutomatismsClonic?	ECS (Ictal BS), EGS-only (1 d)	+ (1 d)	Normal (2.5 y)
6	M	3 y, 3 mo	c.1279G>C, p.(Glu427Gln) homozygous	12 h	BS (2 d), asynch^c^, symm	SequentialMyoclonic/ClonicTonicAutomatisms	EGS-only < 10 s (BRD) (2 d)	Not reported	Mild ED p-z (3 y)
7	M	3 y, 4 mo	c.1279G>C, p.(Glu427Gln); c.834G>A, p.(Val 278=)	4 d	BS (5 d), asynch^c^, symm	SequentialTonicMyoclonic/Clonic	ECS, EGS-only (9 d)	+ (9 d)	Normal (2 y)
8	M	16 y, 9 mo	c.1279G>C, p.(Glu427Gln); c.(650+244_650+ 254)_(695+951_695+ 962)del	6 d	ED L o (8 d)	SequentialTonicClonic/MyoclonicAutomatisms	ECS, EGS-only (8 d)	+ (20 d)	Mild DSBA (17 y)
9	F	25 y	c.1279G>C, p.(Glu427Gln) homozygous	6 h	BS (2 d), asynch^c^, symm	SequentialMyoclonicTonicClonicAutomatismsSpasms?	ECS, EGS-only (1 d)	+ (9 d)	Mild ISBA (26 y)
10	F	25 y	c.1513G>C, p.(Gly505Arg) homozygous	1 d	BS^b^ (2 d), synch	SequentialClonicTonic	ECS (5 d)	+ (3 d)	Mild DSBA (21 y)
11	M	26 y	c.1279G>C, p.(Glu427Gln); c.(650+244_650+ 254)_(695+951_695+ 962)del	2 h	MF ED (1 d)	SequentialTonicClonicAutomatisms	No (PN 2 d)	+ (2 d)	Moderate DSBA (20 y)
12	F	32 y	c.1279G>C, p.(Glu427Gln); c.1513G>C, p.(Gly505Arg)	2 d	ED MF (10 d)	TonicClonic	ECS (10 d)	Not reported	Mild DSBA (26 y)
13	M	55 y	c.1279G>C, p.(Glu427Gln) homozygous	4 h	Alternating bil high ampl delta slow wave (15 d)	TonicClonicMyoclonic?	No (first EEG 15 d)	Not reported	Mild DSBA (55 y)

^a^From Jamali et al. (18).

^b^Amplitudes of suppression periods not specified but described as low voltage.

^c^Bursts with asynchronous elements.

ampl, amplitude; asynch, asynchronous; bil, bilateral; BS, burst suppression; Cont, continuous, d, days; DSBA, diffuse slow background activity; ECS, electroclinical seizures; EGS, electrographic-only seizures; ED, epileptiform discharges; h, hours; ISBA, intermittent slow background activity; L, left; MF, multifocal; o, occipital; p-z, parietal midline; symm, symmetric; synch, synchronous.

All newborns appeared uncomfortable and hyperirritable. Abnormal eye movements, facial grimacing, prominent cries, startle reactions, and jerky movements were common features and clinical-only events without EEG correlate were identified in 10 patients. These features were often difficult to interpret. All patients had respiratory distress; 12 required ventilation support after birth, in several this was possibly related to seizure activity. In Patient 7, there was a clear correlation between recorded focal temporal electrographic seizure activity lasting >1 min and episodes of apnoea and desaturation without other ictal clinical signs. Patient 10 required resuscitation immediately after birth. Seizure breakthrough in older childhood and adult age was rare and presented mainly as tonic-clonic seizures, mostly associated with intercurrent illness or treatment non-adherence (18).

### 3.2 EEG prior to PN exposure

A BS pattern was recorded in eight patients (Patients 2–7 and 9–10) during the first 5 days of life prior to PN treatment (Table 2). In seven, the pattern consisted of alternating periods of marked suppression < 5–10 μV and periods of high voltage bursts (50–250 μV). In one patient (Patient 5), the pattern featured very high (1,000–1,500 μV) voltage bursts. Interburst intervals lasted 1–10 s, and in one (Patient 4) up to 25 s wake and 50–60 s in sleep. Burst intervals predominantly consisted of mixed theta-delta frequencies and multifocal spikes and sharp-waves, lasting 1–10 s. The BS pattern was symmetric, mostly synchronous with asynchronous elements in three patients.

Three of the patients without BS patterns had background activities with alternating amplitudes and episodes of attenuation consistent with excessive discontinuity and interictal epileptiform discharges (IEDs); multifocal and bilateral, and in one focal left occipital region, whereas alternating bilateral high amplitude slow delta background activity was seen in another (Table 2).

**Figure 1:**
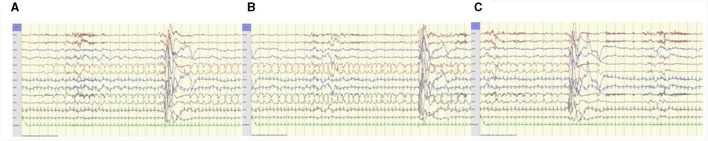
Patient 5, 1 day old. Electrographic seizure-only; **(A)** starts with 1 Hz sharp-waves, **(B)** evolves to rhythmic 1–1.5 Hz sharp-waves and spikes left central region, **(C)** ends abruptly followed by postictal suppression over left hemisphere. ECG artifacts right hemisphere. Anteroposterior bipolar montage; longitudinal (Fp1-T3, T3-O1, Fp2-T4, T4-O2, Fp1-C3, C3-O1, Fp2-C4, C4-O2), transverse (T3-C3, C3-Cz, Cz-C4, C4-T4); 15 mm/s trace speed (1 s between vertical bars); 100 μV/cm; low-pass filter 70 Hz; high-pass filter 0.50 Hz; notch filter 50 Hz.

Electroclinical seizures occurred in 10 patients; two had ictal BS patterns. Electrographic-only seizures were recorded in eight patients (Figure 1); in two lasting < 10 s [previously described as brief rhythmic discharges (BRDs)]. Electrographic-only seizure activity was mostly focal side-shifting runs of rhythmic sharply contoured activity and intermixed spikes and sharp waves with evolving patterns. A desynchronization pattern was rare and only occurred in one patient (Patient 5). Hypsarrhythmia patterns were absent.

### 3.3 EEG during PN trial

Twelve of the 13 patients underwent a trial bolus of intravenous (i.v.) PN (Table 3). Patient 5 started peroral B6 treatment in the form of pyridoxal 5′-phosphate on day 18 (18), but switched to PN when the genetic variant was identified on day 24. EEG recordings were available in all these patients prior to the first B6 exposure, in 11 during PN injection and during follow up (Table 3). The oldest patient (55 y) had no recordings prior to or during the trial.

**Table 3:** EEG and clinical responses to B6 exposure and follow-up EEG findings.

**Patient no**.	**Age at PN trial**	**EEG during PN trial**			**Clinical effect**	**ASM at PN trial**	**EEG follow up, 1st month, days after PN trial (+ d)**

		**Type of recording**	**Pattern at start**	**Effect**			
**Improved EEG during PN trial**				
1	2 h	aEEG	ED bil	Improvement	Response (< 1 h)	None	NA
2	2 d	Standard	BS	Improvement^a^	Seizure free at start and during trial	PB	Normal (+8 d)
10	3 d	LTM	BS	Improvement^a^	Prompt^b^	DZP, PB, PHT	Normal (+5 d)
**Unchanged EEG during PN trial**				
3	2.5 mo	LTM	BS	None	None	CLZ, DZP, LDC, PB, VAL	Unchanged (+6 d)
4	6 d	Standard	BS	None	Delayed (< 1 d)	LEV, PB	Improved (+6 d)
6	2 d	aEEG	BS	None	Uncertain	LEV, MDZ, PB, PHT	Unchanged (+1 d)Improved (+3 d)
7	5 d	LTM	BS	None	Seizure free at start and during trial	CBZ, LEV, MDZ, PB, PHT	Improved (+1 d)Normal (+2 d)
8	20 d	Standard, LTM	ED L o	None	Seizure increase	CLZ, MDZ	Normal (+14 d)
9	21 d	Standard	ED bil asynch	None	Prompt^b^	CBZ, LDC, PB, PHT	Improved (+10 d)
**Deterioration of EEG during PN trial**				
11	2 d	Standard	ED MF	Increased ED	Prompt^b^	BDZ, PB, PHT	Improved (+5 d)
12	11 d	aEEG	ED	Conversion to BS	Prompt^b^	DZP, PB, PHT	Normal (+20 d)
**No PN trial/no EEG during PN trial**				
5	No PN trial, but peroral PLP from 18 d	NA	NA	NA	NA (PLP effect; PN from 24 d)	CLZ, MDZ, TPM, VPA	Unchanged (+4 d)Improved (+8 d)
13	2 d	None	NA	NA	Prompt^b^	DZP, PB	Alternating bil high ampl delta slow wave (+15 d)Normal (+18 d)

^a^Less epileptiform elements and gradually more continuous background activity.

^b^Defined as seizure control within 3 min.

ampl, amplitude; ASMs, Antiseizure medications; asynch, ascynhronous; bil, bilateral; BS, Burst suppression; CBZ, carbamazepine; CLZ, clonazepam; d, day; DZP, diazepam; ED, Epileptiform Discharges; h, hour; L, left; LEV, levetiracetam; LDC, lidocaine; LTM, long-term monitoring; MDZ, midazolam; mo, month; MF, multifocal; NA, not applicable; o, occipital; PB, phenobarbital; PHT, phenytoin; PLP, pyridoxal 5 phosphate; PN, Pyridoxine; TPM, topiramate; VPA, valproate.

**Figure 2:**
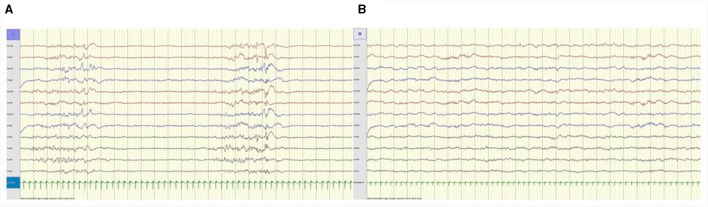
**(A)** Patient 2, 3 days old. Awake EEG tracing prior to PN trial shows burst suppression pattern. **(B)** Same patient, 40 min after PN i.v. EEG tracing shows improvement, resolution of epileptiform discharges and continuous background activity. Anteroposterior bipolar montage; longitudinal (Fp1-T3, T3-O1, Fp2-T4, T4-O2, Fp1-C3, C3-O1, Fp2-C4, C4-O2), transverse (T3-C3, C3-Cz, Cz-C4, C4-T4); 15 mm/s trace speed (1 s between vertical bars); 100 μV/cm; low-pass filter 70 Hz; high-pass filter 0.50 Hz; notch filter 50 Hz.

Median delay from first seizure to first PN injection was 2 days (IQR 5.5; range 0 d-2.5 mo). Immediate EEG improvement was seen in three, two with clear clinical response (Figure 2); the third had no clinical seizures at time of trial, but experienced sustained seizure control with continued peroral PN treatment (Table 3).

Six patients showed no apparent change of paroxysmal activity; one had prompt clinical effect, one had delayed effect (< 1 day), one had no effect, one had uncertain effect, and another had more seizures (Table 3). The duration of EEG recordings after i.v. PN was variable; six patients had >24 h continuous EEG recordings (Patients 1, 3, 6, 8, 10 and 12), two between 30–60 min (Patients 2 and 4), two for 5–10 min (Patient 7 and 9) and not reported in another (Patient 11). In summary, all three patients with improved EEG during PN trial had good clinical effect or non-recurrence of seizure activity.

Two patients had increased paroxysmal activity, one as a conversion to burst suppression. Both experienced prompt clinical effect.

EEG flattening after PN trial was not seen. Adverse reactions in the form of respiratory depression were not reported.

### 3.4 EEG follow up after PN exposure

A delayed effect was common. EEG taken 2–20 days after PN trials showed marked improvement in 10 of 12 patients, six of them had normalized background activity. Patient 6 had an initially unchanged EEG pattern but had considerable improvement of EDs after 3 days. In Patient 5 who received peroral pyridoxal 5′-phosphate, the EEG improved within 8 days. Patient 13 had no EEG recording prior to the PN trial. BS resolved within the first 2 weeks of life in seven patients (Patient 2, 4, 5, 6, 7, 9, and 10). In Patient 3 with a failed PDE trial at age 2.5 mo, a modified BS pattern persisted. After 3 months, an alternating bilateral high amplitude slow delta pattern with occasional epileptiform discharges (ED) continued until death in status epilepticus at age 10 mo.

### 3.5 Long-term EEG follow-up

EEG background activity remained relatively unchanged in serial recordings in 10 patients with established PN treatment (*n* = 93) of which 22 recordings were performed in adult age. In children, EEG was normal for long periods (years), but all had occasional mild intermittent or diffuse slowing, sometimes with variable focal elements (Supplementary material). Sporadic EDs limited to the age below 6 years were seen in seven of these patients, whereas in Patient 13, rare EDs occurred along with a variable mild, diffuse slow background abnormality without persistent changes up to the age of 55. Most follow-up EEGs were routine recordings (82%), but some were taken after seizure breakthrough, usually with unchanged findings. However, in Patient 9, EEG was normal for 19 years until the last recording, which showed mild intermittent slowing at age 26.

All subjects aged >16 years had some degree of background slowing at last EEG follow up, whereas three of the younger ones had normal background activity (Table 2, Supplementary material). All children were treated with the lysine-restricted/arginine-enriched diet. No clear EEG improvement was evident after dietary intervention in seven children, compared to the evolution of EEG findings in patients not receiving the diet (Supplementary material).

### 3.6 Neuroimaging

Brain MRI abnormalities raised the suspicion of alternative causes of epilepsy in some patients (18). Patient 5 had extensive early vascular abnormalities (hemorrhages, venous thrombosis). Patients 8 and 12 had birth asphyxia and displayed diffuse substance loss and ventriculomegaly in later childhood/adult age. They were left with CP; in Patient 12 with severe developmental delay and quadriplegia. A hypoplastic or thinned corpus callosum was seen in five subjects (Patients 1, 5, and 8–10). MRI abnormalities corresponded poorly to EEG findings (18).

## 4 Discussion

### 4.1 Early EEG findings

The present study provides important information regarding the role of EEG in the diagnostic procedures of PDE-ALDH7A1. Early EEG abnormalities were abundant and occurred in the form of bilateral, multifocal and focal epileptiform discharges, as well as BS patterns. Our study demonstrates the shortcomings of an initial empirical assessment based on clinical and EEG response to a single PN challenge in neonates with PDE and seizures resistant to ASMs. The findings suggest that failed or ambiguous PN trials have contributed to previous under-recognition of PDE. This study confirms that a burst suppression EEG pattern is common in PDE and may trigger the suspicion of this diagnosis. However, underlying pathological factors for the development of the BS abnormality are diverse (12, 14, 24). A systematic review of the relationship between neonatal seizures and etiology demonstrated significant associations between burst suppression and all categories of genetic disorders and between unspecified vitamin-related disorders and multifocality (25). Moreover, asynchronous BS patterns have been found to be more common in genetic and/or metabolic etiologies (26).

BS is associated with early infantile DEEs (1) and various other etiologies including comatose states, hypothermia and anesthesia. The underlying neurophysiological mechanisms of BS are incompletely understood (27, 28). Asymmetric and asynchronous BS patterns have been reported in patients with corpus callosum abnormalities such as in Aicardi syndrome (29), suggesting that corpus callosum may be involved in the synchronization of this phenomenon (30). Interestingly, corpus callosum hypoplasia is a common feature of PDE-ALDH7A1. Of the five patients with corpus callosum abnormalities (18), BS was recorded in three. Based on prominent BS patterns, we speculate whether some patients with DEEs previously labeled as Ohtahara syndrome/Early myoclonic encephalopathy (31, 32) may have been unrecognized PDE. In these conditions, early mortality is high (32).

### 4.2 Pitfalls of PN trials

Of the 11 subjects undergoing initial PN trials with simultaneous EEG recordings, the immediate responses were variable and sometimes misleading (Table 3). Seizure control and EEG findings did not always correlate. Previous studies have emphasized that the early EEG response to PN may be ambiguous, as attenuation of multifocal sharp or spike complexes and burst suppression may be delayed; it may take several hours to 5 days or even longer for the EEG to show normal background rhythms (1, 10, 11, 33, 34). Moreover, the clinical assessment is hampered by a multitude of epileptic and non-epileptic manifestations within the setting of neonatal seizures. Seizure manifestations are categorized as “electroclinical” when clinical events and ictal activity correlate, and “electrographic-only” when clinical symptoms are lacking. “Clinical-only” events without EEG correlate are considered non-epileptic in nature and may represent exaggerated reflex behavior or non-specific motor phenomena mimicking epileptic seizures. Thus, ictal symptoms in the neonate should have an EEG signature to be identified as true epileptic seizures (19, 35). Electrographic-only seizures occurred in eight patients. The phenomenon of electro-clinical uncoupling is thought to be facilitated by the effect of ASMs, particularly demonstrated for phenobarbital and phenytoin (19, 36), and all these patients received conventional ASM treatment, mostly including these two drugs (Table 3).

Of the five patients with prompt seizure control from PN (Patients 1 and 9–12), only two (Patients 1 and 10) showed immediate EEG improvement (Table 3). In one (Patient 9), the EEG was unchanged and in two EEG deteriorated; in Patient 11, increased epileptiform activity was observed, and in Patient 12, conversion to a burst suppression pattern took place, a phenomenon which previously has been reported (10, 12).

In two patients with delayed clinical effect within 1 day (Patient 4) or with uncertain effect (Patient 6), the EEG remained unchanged for >24 h. In Patient 8, who initially had seizure increase, the EEG was also unchanged. Following the first failed PN trial, he was considered to have a transient effect of phenytoin. Another PN trial after 12 days had some clinical effect, and he continued PN along with levetiracetam due to persistent sporadic seizures (18). It has been suggested that some patients with incomplete seizure control with PN alone may have additional causes of epilepsy that require supplementary treatment with traditional ASMs (37). Improved diagnostics by biomarkers and genetic tests provide new insights into the phenotypic spectrum of PDE-ALDH7A1, including cases with only partial responsiveness to PN.

According to present experience, traditional i.v. PN trials are not reliable; the clinical effect may be delayed and difficult to assess and poor immediate EEG response or even deterioration does not exclude the diagnosis. Improvement of EEG in response to PN administration is also reported in some cases of non-PDE infantile seizures (16–18, 33, 38). Recent recommendations on the management of neonatal seizures state that the diagnosis of PDE is uncovered by PN injections with EEG monitoring the response (39). Where available, biomarker/rapid genetic testing should be performed (5) since EEG response may be deferred or initially even paradoxical. Nevertheless, a PN trial (i.v. or peroral) should remain as the first treatment attempt at the least suspicion of the diagnosis. Despite apparent unresponsiveness, PN should be continued until ultimate results from molecular genetic testing are available (5, 40, 41). If genetic testing is unavailable, PN should continue for at least 3–5 days before concluding it is not effective (40). EEG monitoring remains essential for identifying both electrographic-only seizures, isolated ictal apnoea and clinical-only events, allowing for a more accurate assessment of the true seizure burden.

As reported in our previous article on the clinical aspects of this cohort, the birth prevalence of diagnosed ALDH7A1-related epilepsy appears to be increasing in Norway (18). All adult patients (>18 y) were identified by prompt clinical response to the first PN exposure during early infancy, whereas the clinical effect in the younger part of the cohort often was delayed or uncertain. In children, the diagnoses were early confirmed by pathogenic *ALDH7A1* variants. The confusing variability and discordance of clinical and EEG responses to PN trials in the present study highlights the diagnostic problems, which may have resulted in previous underdiagnosis of this rare condition.

There are few reports on untreated patients with early seizure onset PDE-ALDH7A1. Patient 3, the elder brother of Patient 10, underwent an unsuccessful PN trial during status epilepticus at age 2.5 mo, and died more than 25 years ago at age 10 mo without receiving PN treatment. It is unknown whether more PDE patients in Norway, even recent ones, may have been disregarded for genetic testing due to failed clinical and EEG responses to PN according to prevailing clinical diagnostic procedures. Early identification of the disorder is critical for survival and prognosis.

### 4.3 Respiratory distress

Respiratory distress was the presenting symptom in all patients, even after normal birth. All except one, needed postnatal respiratory support. Patient 8 had subtle serial seizures with severe desaturations (18). In Patient 7, serial focal temporal ictal electrographic activity > 1 min corresponded to episodes of apnoea in the absence of motor seizures. Autonomic nervous system manifestations, including variation in respiratory rate and vasomotor and heart rate changes are recognized ictal phenomena in the neonate (19, 42). The present EEG findings confirm that respiratory failure in PDA-ALDH7A1 may be related to ictal activity. However, the suggestion of a deep limbic/paralimbic mesial temporal cortex breathing modulation network as a substrate for ictal apnoea (43) underlines the limitations of the scalp EEG to detect such ictal events (42). Moreover, a patient with post-mortem confirmed PDE-ALDH7A1, who died at age 15 h from rapid onset respiratory failure without obvious seizure manifestations, has recently been reported (44).

Our findings corroborate that newborns with PDE-ALDH7A1 are susceptible to respiratory failure. Neonatal asphyxia with lactic acidosis is strikingly common (18, 45). The possibility of ictal apnea should receive attention (42). Six patients experienced early asphyxia, and two were left with cerebral palsy (Patients 8 and 12) (18). Elements of hypoxic-ischemic encephalopathy should never overshadow the consideration of PDE-ALDH7A1.

### 4.4 EEG follow-up

EEG background activity remained relatively unchanged in the follow-up EEGs in patients with established PN treatment, including those performed in adult age. Most were routine recordings, but some were performed after seizures, often related to PN discontinuation or non-adherence as well as intercurrent illness (18). Mild unspecific slowing occurred in early childhood in several recordings irrespective of recent seizures, although in some, mild transient postictal changes were found. Sporadic epileptiform discharges occurred in early childhood and throughout adult age in the oldest patient. Unfortunately, our small and heterogenous material does not allow for a further meaningful systematic investigation of the EEG evolution in various age groups. Nevertheless, all five adult patients were left with elements of slowed background activity, which remained fairly stable over time. Details are given in the Supplementary material. We conclude that EEG did not exhibit obvious progressive changes over time.

PN only counteracts the functional vitamin deficiency and does not abolish the underlying metabolic disturbance causing continuous exposure to potentially neurotoxic metabolites (7). In spite of the fact that cognitive function appeared to be stable (18) with no clear emergent EEG changes suggesting progressing encephalopathy in adult life (46), it is unknown whether dietary interventions with lysine restriction/arginine enrichment might be of benefit in adulthood, or whether this effect is limited to the developing brain. In adult patients, long-term studies with standardized follow-up procedures including EEG recordings are, therefore, warranted. Unfortunately, no systematic EEG follow-up before and after dietary intervention was performed in this retrospective study, precluding meaningful comparisons of possible effects on the EEG activity.

## 5 Limitations and strengths

As emphasized in the recent publication on the clinical aspects of the PDE-ALDH7A1 cohort (18), this type of retrospective study has shortcomings that also apply to the present study focussing on EEG changes. However, a major strength is the close and willing cooperation between the various hospital-based EEG services in Norway that facilitated this type of national survey. All relevant EEG laboratories at seven Norwegian hospitals were contacted, and all available EEG data spanning a period of 55 years were assessed. Equipment, montages, protocols and the duration of saved segments varied between hospitals and time of recordings. Seventy-eight were digital recordings while in 37, only written reports were available (Table 1). Their quality was variable due to inconsistent reporting of EEG patterns; nevertheless, they provided essential elements for analysis. In three patients, recordings during PN trials were limited to amplitude integrated EEG (aEEG). The interpretation of longitudinal EEG data were partly hampered by the various situations in which recordings were performed, particularly in children (e.g., recent seizures and treatment non-adherence etc.).

We also acknowledge limitations concerning the clinical assessments of the seizure burden in neonates due to the common occurrence of non-epileptic clinical-only events as well as the phenomenon of electrographic-only seizures. Moreover, clinical seizure classifications according to the current modified ILAE framework for neonatal seizures may have been imprecise, as they were based on interpretations from medical record descriptions of variable accuracy, particularly with scarce details in the oldest patients. Unfortunately, ictal video EEGs were not available for this study.

## 6 Conclusion

The present study confirms that a burst suppression EEG pattern in neonatal seizures should raise the suspicion of PDE-ALDH7A1 and that respiratory distress is common in newborns with this disorder and may be related to subtle seizures. We show that the EEG response during the first PN trial is variable and may be delayed. However, neither ambiguous clinical effect nor initial EEG unresponsiveness should defer treatment with PN, which should be continued until results from biomarkers and genetic testing are available. Reliance on a single PN trial may result in diagnostic delay and sustained under-recognition of this readily treatable condition. Lastly, while an apparently unchanged cognitive function and stable or only slightly abnormal EEG background activity beyond childhood age weigh against a progressive metabolic encephalopathy; further studies on dietary treatment in adult life are warranted.

## Data availability statement

The raw data supporting the conclusions of this article will be made available by the authors, without undue reservation.

## Ethics statement

The studies involving humans were approved by the Regional Ethical Committee of Mid-Norway (No. 200284). The studies were conducted in accordance with the local legislation and institutional requirements. Written informed consent for participation in this study was provided by the participants' legal guardians/next of kin. Written informed consent was obtained from the individual(s), and minor(s)' legal guardian/next of kin, for the publication of any potentially identifiable images or data included in this article.

## Author contributions

VA: Writing—original draft, Writing—review & editing, Conceptualization, Data curation, Formal analysis, Funding acquisition, Investigation, Methodology, Project administration, Validation. AJ: Writing—review & editing, Conceptualization, Data curation, Investigation, Methodology. AS: Writing—review & editing, Conceptualization, Data curation, Investigation. EK: Writing—review & editing, Conceptualization, Data curation, Investigation. TT: Writing—review & editing, Conceptualization, Data curation, Investigation. GK: Writing—review & editing, Data curation, Investigation. SS: Writing—review & editing, Data curation, Investigation. LB: Writing—review & editing, Data curation, Supervision. TS: Writing—review & editing, Data curation, Supervision, Validation. EB: Data curation, Supervision, Writing—review & editing, Conceptualization, Formal analysis, Investigation, Methodology, Project administration, Validation, Writing—original draft.

## References

[1] Zuberi SM Wirrell E Yozawitz E Wilmshurst JM Specchio N Riney K. ILAE classification and definition of epilepsy syndromes with onset in neonates and infants: position statement by the ILAE Task Force on Nosology and Definitions. Epilepsia. (2022) 63:1349–97. 10.1111/epi.17239 35503712

[2] Gospe SM Jr. Pyridoxine-dependent epilepsy. In:Adam MP Feldman J Mirzaa GM Pagon RA Wallace SE Bean LJH et al., editors. ALDH7A1. Seattle, WA: GeneReviews (R). p. 1993–2023. 20301659

[3] Hunt AD Jr Stokes J Jr Mc CW Stroud HH. Pyridoxine dependency: report of a case of intractable convulsions in an infant controlled by pyridoxine. Pediatrics. (1954) 13:140–5. 10.1542/peds.13.2.140 13133562

[4] Mills PB Struys E Jakobs C Plecko B Baxter P Baumgartner M. Mutations in antiquitin in individuals with pyridoxine-dependent seizures. Nat Med. (2006) 12:307–9. 10.1038/nm1366 16491085

[5] Coughlin CR Tseng LA Abdenur JE Ashmore C Boemer F Bok LA. Consensus guidelines for the diagnosis and management of pyridoxine-dependent epilepsy due to alpha-aminoadipic semialdehyde dehydrogenase deficiency. J Inherit Metab Dis. (2021) 44:178–92. 10.1002/jimd.12332 33200442

[6] Plecko B. On pathways and blind alleys—the importance of biomarkers in vitamin B(6)-dependent epilepsies. J Inherit Metab Dis. (2023) 46:839–47. 10.1002/jimd.12655 37428623

[7] Coughlin CR Tseng LA Bok LA Hartmann H Footitt E Striano P. Association between lysine reduction therapies and cognitive outcomes in patients with pyridoxine-dependent epilepsy. Neurology. (2022) 99:e2627–36. 10.1212/WNL.0000000000201222 36008148 PMC9754645

[8] Tseng LA Hoytema van Konijnenburg EMM Longo N Andrews A van Wegberg A Coene KLM. Clinical reasoning: pediatric seizures of unknown cause. Neurology. (2022) 98:1023–8. 10.1212/WNL.0000000000200711 35470136

[9] Nabbout R Soufflet C Plouin P Dulac O. Pyridoxine dependent epilepsy: a suggestive electroclinical pattern. Arch Dis Child Fetal Neonatal Ed. (1999) 81:F125–9. 10.1136/fn.81.2.F125 10448181 PMC1720985

[10] Naasan G Yabroudi M Rahi A Mikati MA. Electroencephalographic changes in pyridoxine-dependant epilepsy: new observations. Epileptic Disord. (2009) 11:293–300. 10.1684/epd.2009.0280 20031502

[11] Jiao X Xue J Gong P Wu Y Zhang Y Jiang Y. Clinical and genetic features in pyridoxine-dependent epilepsy: a Chinese cohort study. Dev Med Child Neurol. (2020) 62:315–21. 10.1111/dmcn.14385 31737911

[12] Schmitt B Baumgartner M Mills PB Clayton PT Jakobs C Keller E. Seizures and paroxysmal events: symptoms pointing to the diagnosis of pyridoxine-dependent epilepsy and pyridoxine phosphate oxidase deficiency. Dev Med Child Neurol. (2010) 52:e133–42. 10.1111/j.1469-8749.2010.03660.x 20370816

[13] Mikati MA Trevathan E Krishnamoorthy KS Lombroso CT. Pyridoxine-dependent epilepsy: EEG investigations and long-term follow-up. Electroencephalogr Clin Neurophysiol. (1991) 78:215–21. 10.1016/0013-4694(91)90035-3 1707793

[14] Falsaperla R Vari MS Toldo I Murgia A Sartori S Vecchi M. Pyridoxine-dependent epilepsies: an observational study on clinical, diagnostic, therapeutic and prognostic features in a pediatric cohort. Metab Brain Dis. (2018) 33:261–9. 10.1007/s11011-017-0150-x 29178011

[15] Okumura A. Electroencephalography in neonatal epilepsies. Pediatr Int. (2020) 62:1019–28. 10.1111/ped.14227 32153072

[16] Teune LK vd Hoeven JH Maurits NM Bos AF Alffenaar JW Reijngoud DJ. Pyridoxine induces non-specific EEG alterations in infants with therapy resistant seizures. Seizure. (2007) 16:459–64. 10.1016/j.seizure.2007.02.008 17408982

[17] Bok LA Maurits NM Willemsen MA Jakobs C Teune LK Poll-The Poll-The BT. The EEG response to pyridoxine-IV neither identifies nor excludes pyridoxine-dependent epilepsy. Epilepsia. (2010) 51:2406–11. 10.1111/j.1528-1167.2010.02747.x 20887371

[18] Jamali A Kristensen E Tangeraas T Arntsen V Sikiric A Kupliauskiene G. The spectrum of pyridoxine dependent epilepsy across the age span: a nationwide retrospective observational study. Epilepsy Res. (2023) 190:107099. 10.1016/j.eplepsyres.2023.107099 36731270

[19] Pressler RM Cilio MR Mizrahi EM Moshe SL Nunes ML Plouin P. The ILAE classification of seizures and the epilepsies: modification for seizures in the neonate. Position paper by the ILAE. Task Force on Neonatal Seizures. Epilepsia. (2021) 62:615–28. 10.1111/epi.16815 33522601

[20] Tsuchida TN Wusthoff CJ Shellhaas RA Abend NS Hahn CD Sullivan JE. American clinical neurophysiology society standardized EEG terminology and categorization for the description of continuous EEG monitoring in neonates: report of the American Clinical Neurophysiology Society critical care monitoring committee. J Clin Neurophysiol. (2013) 30:161–73. 10.1097/WNP.0b013e3182872b24 23545767

[21] Kane N Acharya J Benickzy S Caboclo L Finnigan S Kaplan PW. A revised glossary of terms most commonly used by clinical electroencephalographers and updated proposal for the report format of the EEG findings. Revision 2017. Clin Neurophysiol Pract. (2017) 2:170–85. 10.1016/j.cnp.2017.07.002 30214992 PMC6123891

[22] Andre M Lamblin MD d'Allest AM Curzi-Dascalova L Moussalli-Salefranque F. Electroencephalography in premature and full-term infants. Developmental features and glossary. Neurophysiol Clin. (2010) 40:59–124. 10.1016/j.neucli.2010.02.002 20510792

[23] Beniczky S Aurlien H Brogger JC Hirsch LJ Schomer DL Trinka E. Standardized computer-based organized reporting of EEG: SCORE - Second version. Clin Neurophysiol. (2017) 128:2334–46. 10.1016/j.clinph.2017.07.418 28838815

[24] Plecko B Paul K Mills P Clayton P Paschke E Maier O. Pyridoxine responsiveness in novel mutations of the PNPO gene. Neurology. (2014) 82:1425–33. 10.1212/WNL.0000000000000344 24658933 PMC4001193

[25] Nunes ML Yozawitz EG Zuberi S Mizrahi EM Cilio MR Moshe SL. Neonatal seizures: Is there a relationship between ictal electroclinical features and etiology? A critical appraisal based on a systematic literature review. Epilepsia Open. (2019) 4:10–29. 10.1002/epi4.12298 30868112 PMC6398099

[26] Yang H Gong P Jiao X Zhou Q Zhang Y Jiang Y. The relationship between the characteristics of burst suppression pattern and different etiologies in epilepsy. Sci Rep. (2021) 11:15903. 10.1038/s41598-021-95040-4 34354098 PMC8342459

[27] Amzica F. Basic physiology of burst-suppression. Epilepsia. (2009) 50(Suppl.12):38–9. 10.1111/j.1528-1167.2009.02345.x 19941521

[28] Shanker A Abel JH Schamberg G Brown EN. Etiology of burst suppression EEG patterns. Front Psychol. (2021) 12:673529. 10.3389/fpsyg.2021.673529 34177731 PMC8222661

[29] Aicardi J. Aicardi syndrome. Brain Dev. (2005) 27:164–71. 10.1016/j.braindev.2003.11.011 15737696

[30] Lambrakis CC Lancman ME Romano C. Asynchronous and asymmetric burst-suppression in a patient with a corpus callosum lesion. Clin Neurophysiol. (1999) 110:103–5. 10.1016/S0013-4694(98)00102-3 10348328

[31] Fusco L Pachatz C Di Capua M Vigevano F. Video/EEG aspects of early-infantile epileptic encephalopathy with suppression-bursts (Ohtahara syndrome). Brain Dev. (2001) 23:708–14. 10.1016/S0387-7604(01)00280-7 11701283

[32] Ohtahara S Yamatogi Y. Epileptic encephalopathies in early infancy with suppression-burst. J Clin Neurophysiol. (2003) 20:398–407. 10.1097/00004691-200311000-00003 14734930

[33] Fortin O Christoffel K Kousa Y Miller I Leon E Donoho K. Pearls & Oy-sters: delayed response to pyridoxine in pyridoxine-dependent epilepsy. Neurology. (2023) 101:e1828–e32. 10.1212/WNL.0000000000207829 37580162 PMC10634650

[34] Mastrangelo M Gasparri V Bernardi K Foglietta S Ramantani G Pisani F. Epilepsy phenotypes of vitamin B6-dependent diseases: an updated systematic review. Children. (2023) 10:30553. 10.3390/children10030553 PMC10047402 36980111

[35] Shellhaas RA. Neonatal seizures reach the mainstream: the ILAE classification of seizures in the neonate. Epilepsia. (2021) 62:629–31. 10.1111/epi.16857 33634848

[36] Scher MS Alvin J Gaus L Minnigh B Painter MJ. Uncoupling of EEG-clinical neonatal seizures after antiepileptic drug use. Pediatr Neurol. (2003) 28:277–80. 10.1016/S0887-8994(02)00621-5 12849880

[37] Basura GJ Hagland SP Wiltse AM Gospe SM Jr. Clinical features and the management of pyridoxine-dependent and pyridoxine-responsive seizures: review of 63 North American cases submitted to a patient registry. Eur J Pediatr. (2009) 168:697–704. 10.1007/s00431-008-0823-x 18762976

[38] Cirillo M Venkatesan C Millichap JJ Stack CV Nordli DR Jr. Case report: intravenous and oral pyridoxine trial for diagnosis of pyridoxine-dependent epilepsy. Pediatrics. (2015) 136:e257–61. 10.1542/peds.2014-2423 26101365

[39] Yozawitz E. Neonatal seizures. N Engl J Med. (2023) 388:1692–700. 10.1056/NEJMra2300188 37133587

[40] Pressler RM Abend NS Auvin S Boylan G Brigo F Cilio MR. Treatment of seizures in the neonate: guidelines and consensus-based recommendations–special report from the ILAE Task Force on Neonatal Seizures. Epilepsia. (2023) 64:2550–70. 10.1111/epi.17745 37655702

[41] Mastrangelo M Cesario S. Update on the treatment of vitamin B6 dependent epilepsies. Expert Rev Neurother. (2019) 19:1135–47. 10.1080/14737175.2019.1648212 31340680

[42] Falsaperla R Consentino MC Vitaliti G Marino S Ruggieri M. Isolated ictal apnea in neonatal age: clinical features and treatment options. A systematic review. Auton Neurosci. (2022) 243:103034. 10.1016/j.autneu.2022.103034 36174277

[43] Lacuey N Hampson JP Harper RM Miller JP Lhatoo S. Limbic and paralimbic structures driving ictal central apnea. Neurology. (2019) 92:e655–69. 10.1212/WNL.0000000000006920 30635481 PMC6382368

[44] Aquilano G Linner A Ygberg S Stodberg T Henckel E. Case report: fatal outcome of pyridoxine-dependent epilepsy presenting as respiratory distress followed by a circulatory collapse. Front Pediatr. (2022) 10:940103. 10.3389/fped.2022.940103 35967578 PMC9366515

[45] van Karnebeek CD Tiebout SA Niermeijer J Poll-The Poll-The BT Ghani A Coughlin CR 2nd. Pyridoxine-dependent epilepsy: an expanding clinical spectrum. Pediatr Neurol. (2016) 59:6–12. 10.1016/j.pediatrneurol.2015.12.013 26995068

[46] Rayi A Mandalaneni K. Encephalopathic EEG Patterns. Treasure Island: StatPearls (2023). 33232041

