# Supplementary material for: Utility and limitations of EEG in the diagnosis and management of ALDH7A1-related pyridoxine-dependent epilepsy. A retrospective observational study
Source: Front Neurol. 2024 Feb 14;15:1355861. doi: 10.3389/fneur.2024.1355861 (PMC10899485; doi:10.3389/fneur.2024.1355861)
Supplement: Supplementary file 1 [file Data_Sheet_1.pdf]

Supplementary file

Overview of longitudinal EEG background and epileptiform activity in subjects < 16 y on established pyridoxine treatment

Patient number	Main EEG characteristics (age)	Clinical situation
2	1. Normal (10 d)	Routine
4	1. Moderate DSBA, rare ED, L t-o (13 d)<br>*Dietary treatment (3 w)*<br>2. Mild DSBA, rare ED, bil t-o (2.5 mo)<br>3. Rare ED, t-c-p (3 mo)<br>4. Mild DSBA, ED, async bil t-p (1 y)<br>5. Mild ISBA (1.5 y)	Routine<br><br>*1 d after seizure due to fever/intercurrent illness*<br>*Suspected seizures/abnormal movements*<br>Routine<br>Routine
5	1. Mild DSBA, MF/bil ED (27 d)<br>2. Mild DSBA, MF/bil ED (31 d)<br>3. Mild DSBA, MF/bil ED (1 mo)<br>4. Mild ISBA L (2 mo)<br>*Dietary treatment (3 mo)*<br>5. Normal (7 mo)<br>6. Moderate ED async bil f-c (1.5 y)<br>7. Mild ED bil async f-c (2 y)<br>8. Normal (2.5 y)	Routine<br>Routine<br>Routine<br>Routine<br><br>Routine<br>*1 d after seizure without known precipitant*<br>Routine<br>Routine
6	1. Marked DSBA (7 d)<br>2. Mild DSBA (14 d)<br>3. Mild DSBA (1.5 mo)<br>*Dietary treatment (2 mo)*<br>4. Normal (4 mo)<br>5. Normal (9 mo)<br>6. Normal (1.5 y)<br>7. Frequent ED p-z (3 y)<br>8. Mild ED p-z in sleep (3 y)	Routine<br>Routine<br>Routine<br><br>*6 d after seizure due to fever/intercurrent illness*<br>*Seizure due to fever/intercurrent illness*<br>*Seizure due to fever/intercurrent illness*<br>Routine<br>Routine
7	*Dietary treatment (5 w)*<br>1. Normal (1.5 y)	 Routine
8	1. Normal (2 mo)<br>2. Mild DSBA, freq ED async bil (11 mo)<br>3. Mild DSBA, freq ED async bil (1 y)<br>4. Mild DSBA, freq ED async bil (1 y)<br>5. Mild DSBA, freq ED L (1.5 y)<br>6. Mild DSBA, mod ED L (1.5 y)<br>7. Mild DSBA, mod ED f-c, ECS 1 min PER (2 y)	Routine<br>Routine<br>Routine<br>Routine<br>Routine<br>Routine<br>*Seizure due to fever/intercurrent illness*

	8. Mild DSBA, ED R c-p, PER (2 y)	Routine
	9. Mild DSBA, freq ED bil async max R c-p, ECS (2.5 y)	*Seizures without known precipitant*
	10. Moderate DSBA (3 y)	Routine
	11. Mild DSBA, freq ED bil async (5.5 y)	Routine
	Dietary treatment (10 y)	
	12. Mild DSBA (14 y)	Routine
9	1. Mild DSBA (1 mo)	Routine
	2. Mild DSBA (1.5 mo)	Routine
	3. Normal (6 mo)	Routine
	4. Rare ED bil t (9 mo)	*1 day after seizure due to fever/intercurrent illness*
	5. Normal (10 mo)	Routine
	6. Normal (1.5 y)	Routine
	7. Normal (6 y)	Routine
10	1. Normal (1 y)	*Seizure due to fever/intercurrent illness*
	2. Normal (1.5 y)	Routine
	3. Normal (2 y)	Routine
	4. Normal (2 y)	Routine
	5. Normal (3.5 y)	Routine
	6. Moderate DSBA, MF ED (4.5 y)	Routine
11	1. Mild MF ED (16 d)	Routine
	2. Mild Alt high amp SBA (10 mo)	Routine
	3. Normal (1 y)	*2 d after seizure due to fever/intercurrent illness*
12	1. Normal (1 mo)	Routine
	2. Normal (1.5 mo)	Routine
	3. Normal (2 mo)	Routine
	4. Normal (6 mo)	Routine
	5. Normal (1 y)	Routine
	6. Normal (4 y)	Routine
	7. Normal (4 y)	Routine
13	1. Moderate Alt high amp SBA (15 d)	Routine
	2. Normal (3 m)	Routine
	3. Marked DSBA (8 mo)	*1 d after seizure due to treatment discontinuation*
	4. Moderate DSBA (10 mo)	Routine
	5. Normal (1.5 y)	*2 d after seizure due to treatment discontinuation*
	6. Mild DSBA, rare ED R t-p (6 y)	Routine
	7. Normal (8 y)	Routine
	8. Marked DSBA, max R t (8 y)	*3 d after seizure due to GE/fever/PN interruption*
	9. Moderate DSBA, max R f-t (8 y)	Routine
	10. Normal (8 y)	Routine
	11. Mild SW R f-t, rare ED R f-t (8 y)	Routine
	12. Moderate DSBA, rare ED bil f-t (12 y)	Routine

	13. Mild DSBA (13 y)	Routine

Alt high amp SBA, alternating high amplitude slow background activity; async, asynchronous; bil, bilateral; c, central; d, day; DSBA, diffuse slow background activity; ECS, electroclinical seizure; ED, epileptiform discharges; freq, frequent; f, frontal; GE, gastroenteritis; ISBA, intermittent slow background activity; L, left; max, maximum; mod, moderate; mo, month; MF, multifocal; o, occipital; p, parietal; p-z, parietal midline; PER, photoepileptiform response; SW, slow waves; t, temporal; y, year

Overview of longitudinal EEG background and epileptiform activity in subjects > 16 y on established pyridoxine treatment.

Patient number	Main EEG characteristics (age)	Clinical situation
8	1. Mild DSBA (17 y)	Routine
9	1. Normal (19 y)<br>2. Mild ISBA (26 y)	Routine<br>Routine
10	1. Mild DSBA (20 y)<br>2. Mild DSBA (21 y)	Routine<br>Routine
11	1. Moderate DSBA (19 y)<br>2. Moderate DSBA (19 y)<br>3. Moderate DSBA (20 y)	Routine<br>Routine<br>Routine
12	1. Mild DSBA (17 y)<br>2. Mild DSBA (20 y)<br>3. Mild DSBA (22 y)<br>4. Mild DSBA (23 y)<br>5. Mild DSBA (24 y)<br>6. Normal (26 y)	Routine<br>Routine<br>*3 d after seizure due to GE/fever/PN interruption*<br>Routine<br>Routine<br>Routine
13	1. Mild DSBA, rare ED async bil f-t (17 y)<br>2. Mild DSBA (29 y)<br>3. Mild DSBA, rare ED async bil f-t (31 y)<br>4. Mild DSBA, rare ED async bil f (33 y)<br>5. Mild DSBA (35 y)<br>6. Mild DSBA, rare ED async bil f (46 y)<br>7. Mild DSBA, rare ED L t (47 y)<br>8. Mild DSBA (55 y)	*Routine*<br>*6 d after SE abroad due to GE/fever/PN interruption*<br>Routine<br>*Seizure without known precipitant (non-adherence?)*<br>Routine<br>Routine<br>Routine<br>Routine

asynch, asynchronous; bil, bilateral; d, days; DSBA, diffuse slow background activity; ED, epileptiform discharges; f, frontal; GE, gastroenteritis; ISBA, intermittent slow background activity; L, left; PN, pyridoxine; SE, status epilepticus; t, temporal; y, years
